# Volatile Organic Compound Identification-Based Tuberculosis Screening among TB Suspects: A Diagnostic Accuracy Study

**DOI:** 10.3390/arm91040024

**Published:** 2023-07-12

**Authors:** Mayank Badola, Anurag Agrawal, Debabrata Roy, Richa Sinha, Avisham Goyal, Narayan Jeet

**Affiliations:** 1 Department of Health and Family Welfare, Government of Uttarakhand, Dehradun 248001, India; mayankbadola022@gmail.com; 2 Department of TB & Chest, Government Doon Medical College, Dehradun 248001, India; dranurag.1992@gmail.com (A.A.); avisham.goyal@gmail.com (A.G.); 3 Department of Community Medicine, Government Doon Medical College, Dehradun 248001, India; droy97@rediffmail.com; 4 Department of General Medicine, Government Doon Medical College, Dehradun 248001, India; drmadhulata@gmail.com

**Keywords:** VOCs, diagnostic accuracy study, breath testing, Mycobacterium tuberculosis

## Abstract

**Highlights:**

**What are the main findings?**

- The test kit showed significantly high sensitivity and specificity as reliability indicators for a potential non-invasive, rapid, cost-effective technique that uses the volatile biomarkers in exhaled breath for the identification of tuberculosis.
- Volatile organic compounds are evidently strong contenders for being potential tuberculosis biomarkers.

**What is the implications of the main findings?**

- The diagnostic accuracy of the Tuberculosis Breath Analyzer was found to be high for TB detection.
- The performance of the tested Tuberculosis Breath Analyzer was found to be comparable in efficiency to that of the TrueNat assay.

**Abstract:**

Tuberculosis (TB) affects a third of the global population, and a large population of infected individuals still remain undiagnosed—making the visible burden only the tip of the iceberg. The detection of tuberculosis in close-proximity patients is one of the key priorities for attaining the Sustainable Development Goals (SDG) of TB elimination by 2030. With the current battery of screening tests failing to cover this need, the authors of this paper examined a simple and inexpensive point-of-care breath analyzer (TSI-3000(I)), which is based on detecting the volatile organic compounds that are emitted from infected cells and released in exhaled breath as a screening tool for the detection of TB. A single-center pilot study for assessing the diagnostic accuracy of the point-of-care Tuberculosis Breath Analyzer was conducted, and it was compared against the WHO-recommended TrueNat assay, which is a rapid molecular test and was also treated as the reference standard in this study. Of the 334 enrolled participants with TB signs/symptoms, 42.51% were TrueNat positive for Mycobacterium tuberculosis. The sensitivity of the Tuberculosis Breath Analyzer was found to be 95.7%, with a specificity of 91.3% and a ROC area of 0.935. The test kit showed considerable/significant high sensitivity and specificity as reliability indicators. The performance of the Tuberculosis Breath Analyzer tested was found to be comparable in efficiency to that of the TrueNat assay. A large cohort-based multicentric study is feasibly required to further validate and extrapolate the results of the pilot study.

## 1. Introduction

Tuberculosis (TB) is the leading cause of infectious death worldwide, especially in most middle-income countries. The incidence of tuberculosis is declining, but the global situation still revealed that, in the year 2021, an estimated 10.6 million people fell ill with TB, which is equivalent to 134 cases/100,000 population. India alone accounts for 28% of the cases detected worldwide [1]. The data suggest that around one in every three TB patients is undiagnosed. Factors such as lack of awareness and resources, poor infrastructure, poor notification, overall negligence including poverty, undernourishment, and social stigma are major challenges for the huge proportion of the cases that remain undiagnosed—thus increasing the hidden burden of the disease in India [2]. Furthermore, India aims to eradicate TB by 2025, i.e., five years before the global sustainable development goal (SDG) of 2030, which seems far from reality given the current detection and treatment rates [3].

The COVID-19 pandemic has adversely affected TB elimination programs and global TB targets. Importantly, the reporting of new cases was essentially disrupted, resulting in an 18% decline in case incidence (i.e., back to the level of 2012), which is far less than the projected estimate. India accounted for 25% of the reduced estimate, whereas a considerable number of countries experienced far higher reductions [4]. Moreover, there was a relatively large diagnostic gap in India as only 18% of the patients notified were tested with rapid tests at the time of diagnosis, and only 54% were bacteriologically confirmed [5]. The gaps in diagnostic testing were due to the following: non-availability of doctors/lab technicians; inadequate knowledge about TB diagnostic tests among health care providers; reluctance of patients to undergo the TB diagnostic tests due to stigma/confidentiality issues; and the sub-optimal engagement of private health facilities for TB control [6].

There is a continuous search for more reliable and efficient techniques that enable effective management decisions. A potential non-invasive technique is the analysis of the volatile biomarkers from exhaled breath; namely, the so-called volatile organic compounds (VOCs). Pulmonary tuberculosis may alter volatile organic compounds (VOCs) in breath because the Mycobacteria and oxidative stress that result from Mycobacterial infection both generate distinctive VOCs [7]. It was reported that several species of Mycobacteria produce VOC metabolites, which act as chemical “fingerprints”. Of the several techniques that are in practice for the collection, detection, and analysis of exhaled VOCs [8,9,10,11], the use of gas chromatography (GC), which is the gold standard for the detection of volatile organic compounds (VOCs), may viably indicate the relevant metabolic dynamics in the lungs or in blood. VOCs are used in diagnosis, prognosis, and as indicators of treatment response, i.e., biomarkers for a spectrum of illnesses and infections, including those of the respiratory system [12]. Other than being rapid, cost-effective, and non-invasive in nature, VOCs are evidently strong contenders for being potential tuberculosis biomarkers. Preliminary studies also highlight its diagnostic potential for the same. The present study was designed as a pilot study to provide a proof of concept and also preliminary estimates for the diagnostic accuracy of a novel point-of-care non-invasive test that is based on detection of the VOC present in the exhaled breath of TB suspects. The study objectives include estimation of the diagnostic accuracy of a VOC-identification-based TB screening tool (Breath Analyzer TSI-3000 (I), Technoscan, Vaughan, Canada) against the gold standard of PCR-based TRUENAT/CBNAAT in terms of sensitivity, specificity, as well as positive and negative predictive values. The authors also aimed to examine the potential socio-behavioral and epidemiological correlates that influenced the test results.

## 2. Materials and Methods

### 2.1. Study Design

A quasi-experimental pilot study was conducted, between July to December 2022, to evaluate the use of the TSI-3000 (I) Breath Analyzer and all of the pulmonary TB suspects at the Pulmonology Out Patient Department [13] of Government Doon Medical College; these patients also constituted the study population.

### 2.2. Sample Size

A convenient sample of 334 consecutive TB suspects were recruited for the study in the Pulmonology OPD of the Government Doon Medical College between July to December 2022.

### 2.3. Study Tools

1. A study instrument for eliciting socio-demographic attributes of the clients, namely, age, gender, marital status, occupation, religion, ethnicity, socio-economic status etc.
2. TSI3000I Breath Analyzer for Index Testing and the TrueNat/CBNAAT Essay

The TSI3000I Breath Analyzer test (index test) [14] is typically simple, rapid, and is an in vitro diagnostic test that aims to improve ease of access and implementation (compared with confirmatory tests), as well as decrease the proportion of patients requiring more expensive confirmatory testing [15]. The TSI3000I Breath Analyzer test is designed to be used in adults and children identified as having symptoms compatible with TB or those who have high-risk factors for any form of active TB.

### 2.4. Inclusion Criteria

Individuals aged 10 years and above suspected of having pulmonary TB were assessed based on the following: symptoms and signs, e.g., cough, sputum production, night sweats, weight loss or hemoptysis; OR a history of known recent exposure to infection; OR chest X-ray abnormalities that were consistent with active pulmonary TB. All the participants who were willing to give informed written consent were included in the study, and patients who were suffering from HIV and any comorbidities were included.

### 2.5. Exclusion Criteria

Pregnant women were considered ineligible, and those who were lost to follow-up cases, had cases with a previous history of Tuberculosis, and those with invalid breath measurements were also excluded.

### 2.6. Implementation Plan

**Figure 1:**
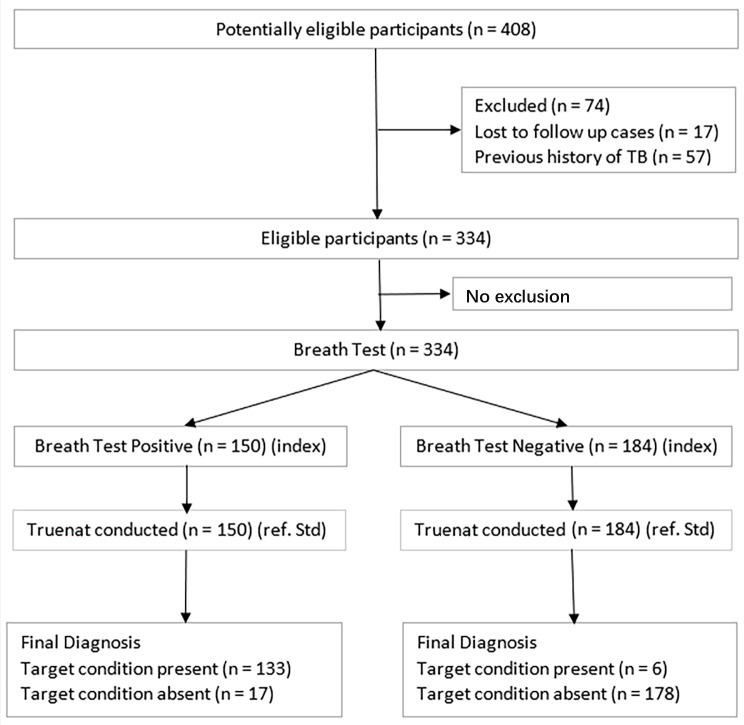
Flow of Patients through the Study.

All TB suspects identified in the studied Pulmonology OPD between July to December 2022 were recruited, and all eligible suspects were offered the index test after obtaining informed consent from the participants (Figure 1). All participants were further subjected to a diagnostic test that was essentially the PCR-based TrueNat/CBNAAT procedure (as per the World Health Organization (WHO) National tuberculosis elimination programme guidelines [16,17]). The clients whose samples were PCR-based TrueNat positive were considered as reference positive subjects (gold standard), and those who were PCR-based TrueNat negative were considered healthy subjects. All the cases reported in the Nikshay TB registry were treated as per the standard protocol. All cases were treated in accordance with national TB guidelines, on a first-line anti-TB treatment on the day of enrolment. Cases with a new diagnosis of HIV infection and those with known HIV co-infection who were not on antiretroviral (ARV) therapy were started, according to national guidelines, on ARV treatment within the first 3 weeks after TB treatment initiation [13].

### 2.7. Point-Of-Care (POC) Breath Test

Breath samples were collected by seven to ten forced expirations onto the sample card, and then the sample card was inserted into the desorber of Tuberculosis Breath Analyzer for data analysis. Once the data were analyzed by the Tuberculosis breath analyzer the results were displayed within 20 s as a Green Screen (TB negative subjects) or a Red Screen (TB positive subjects) on the display panel.

## 3. Results

Of the potentially eligible participants (n = 408), 74 participants were excluded from the statistical analysis of data as the participants did not meet the eligibility criteria.

Table 1 reveals that mean age of the study subjects was 35.8 years, 187 (55.9%) among them were males and 147 (44%) were females; total number of eligible participants was 334 and 139 (41.6%) of them were disease positive; 103 (30.83%) of the study subjects had some lesion present on Chest Xray, 21 (6.28%) each were HIV positive and Diabetics and 34 (10.1%) were currently smoking.

It can be seen from Table 2 that the test kit identified 133 study subjects as disease positive out of a total of 139 study subjects. These disease positives were determined by the gold standard; furthermore, the test kit identified 178 study subjects as disease negative out of the 195 disease negative subjects that were determined by the gold standard.

The Tuberculosis Breath Analyzer identified active pulmonary TB with a sensitivity of 95.7% (95% CI 90.8–94.8%), the specificity was 91.3% (95% CI 86.45–94.8%), the positive predictive value was 88.7%, the negative predictive value was 96.7%, and the ROC area was 0.935. The area under curve (AUC) represents the probability that a random positive subject is positioned to the right of a random negative subject, and the area under ROC curve in the study findings was 0.935, which is very close to 1. This may, therefore, be reasonably assumed that models which are capable of being 100% correct in terms of probability are likely incorrect.

The sub-analysis of the data, as shown in Table 3, showed that the tests showed considerable uniformity in terms of accuracy indicators (sensitivity, specificity, and predictive values) across the different age groups. However, the study analysis shows a relatively low indicator status in the age group of 30–44 years, and as much as 67.2% of the age group of 45–59 were negative for TB; the reasons for this may be due to the patient characteristics of this age group, including difference in smoking, eating, or other such habits that can influence this difference. To prevent interference by VOCs in the environment, compounds such as ethanol and isopropanol were used [18].

**Figure 2:**
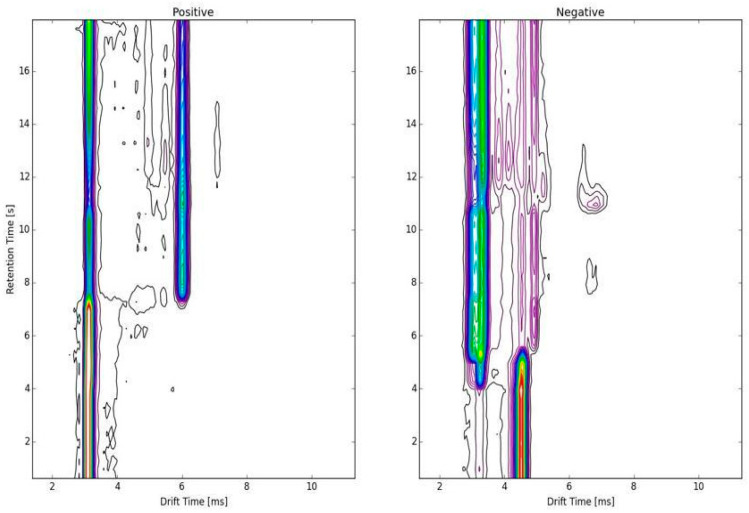
Spectroscopic display/topographic map.

In the spectroscopic display/topographic map of a true positive result in a suspect TB case (Figure 2), every continuous line indicates a peak representative of some chemical substance or fragment of a substance. The brighter the color, the higher the amplitude. The graphs are digital signatures that were deciphered by artificial intelligence. The VOC’s accuracy was not altered or degraded by AI; rather, with the developments in new machine learning and deep learning algorithms, the interpretation will only develop further. Future research can also use neural network models to find deeper associations, allowing for better risk stratification.

## 4. Discussion

With current diagnostic tools, there are several options for reference standards in terms of comparing novel TB diagnostic tools according to STARD guidelines. Each reference standard addresses three categories of participants who are each tested for TB differently: microbiologically confirmed TB (true positive), clinical TB, and no TB (true negative). People with clinical TB may present with symptoms or signs that are suggestive of TB, even though the causative bacterium may not be detectable [19]. These reference standards have associated advantages and disadvantages depending on the target diagnosis and population being studied. For precision in the analysis of the study in context, participants with clinical TB and those who were only syndromically diagnosed were excluded from the analysis.

Breath analysis is attractive because it offers the following: point-of-care location, rapid results without dependency on reagents, non-invasive sampling with a low biosecurity burden, and usability in a range of worldwide scenarios, including low-resourced environments such as community or primary care settings [20].

The apparatus tested in the present study is a Breath Analyzer comprising a fast ion mobility spectrometer (IMS) with a front internal separator unit that works on the thermal vaporization of a breath sample (the samples of which are collected on a nanocarbon-treated sample card). The screening for tuberculosis mainly depends on the WHO-recommended four-symptom screening test (71% sensitivity, 64% specificity), CXR (any abnormality, sensitivity 94%, specificity 89%), and the molecular WHO-recommended rapid diagnostic test (mWDRD) (adult at high risk sensitivity 69%, specificity 99%) [19,21]. The recommended target for a triage test is a sensitivity of 95% and a specificity of 80% [22]. The diagnostic accuracy of the Tuberculosis Breath Analyzer (overall sensitivity and specificity are 95.7% and 91.3%, respectively) showed considerable reliability when compared with the reliability of the TrueNat assay (overall sensitivity and specificity are 85% and 98%, respectively).

The current COVID epidemic has reduced the screening and diagnostics of TB to a large extent, and only an accurate triage test for the initial point of contact will help in surmounting the diagnostic gap.

Several new triage tests, such as the computer-aided detection of X-ray-biomarker-based assays, are under various stages of development as per the FIND tuberculosis diagnostic pipeline, but only one product, ‘e-nose’, (which is based on the detection of volatile organic compounds) has found a degree of success [23,24].

In this study, the Tuberculosis Breath Analyzer test with test kit TSI3000I was the index test, and its results were compared to the WHO-recommended TrueNat assay (which was also used as the reference standard).

An analogous study by Phillips M et al. [25] on symptomatic high-risk subjects found that breath biomarkers identified active pulmonary tuberculosis with a C-statistic (area under curve of receiver operating characteristic) of 0.85 (i.e., 85% overall accuracy, sensitivity = 84.0%, specificity = 64.7%) when the sputum culture, microscopy, and chest radiography were either all positive or all negative. Zetola N.M. [26] et al.—in their study on the diagnosis and assessment of treatment response through analyses of VOCs in breath—estimated the sensitivity of a device at 94.1% (95% confidence interval [CI], 83.8–98.8%) and a specificity at 90.0% (95% CI, 68.3–98.8%) for distinguishing TB cases from controls. In another multicentric study, also conducted in India [12] and which involved a six-minute point-of-care breath test for volatile biomarkers, subjects with active pulmonary TB were identified with an 80% accuracy (area under curve of receiver operating characteristic curve), a sensitivity of 71.2%, and a specificity of 72%. Accuracy increased to 84% in age-matched subgroups. In a population with a 5% prevalence, the breath test was able to identify active pulmonary TB with a 98% negative predictive value and a 13% positive predictive value.

Furthermore, Saktiawati A.M. et al. [27], in a systematic review of 14 studies with 1715 subjects, found the pooled sensitivity and specificity of an electronic nose to be 0.93 (95% CI 0.82–0.97) and 0.93 (95% CI 0.82–0.97), respectively. Moreover, no heterogeneity was found. Those findings corroborate our study findings.

Recommendations: Future studies are warranted to contrast the VOCs against the gold standard with large and diverse sample sizes, and to find its relevance for atypical mycobacteria (MOTT).

Limitations: The current study is a pilot initiative in a single tertiary institution with limited resources. Comparison against gold standards (microbial culture and sputum microscopy) could have been rewarding, but with limited resources, it was compared against a test similar to public health relevance allowing a simpler outcome analysis to justify further use and research for the evolving tolls. Additionally, the feasibility of detection of atypical mycobacteria (MOTT) by VOCs have not been factored in as covering them was beyond the scope of the study. These constraints may limit the study essentially for extrapolation of study results to a larger population.

Comorbidities, age-related, and other related risk factors are undeniable cofounders in this study. Despite these minor limitations, the VOC still appears to be a great screening tool and can help fasten the screening procedures in resource-limited, high-prevalence, and heavy-population settings.

## 5. Conclusions

The diagnostic accuracy of the Tuberculosis Breath Analyzer was found to be high for TB detection. The assays have the potential to be used as a POC TB triage test. In view of the limitations for the extrapolation of results, we recommend further multicentric studies with a larger sample size (ideally in a point-of-care institution such as a primary care center) and to compare the results with a gold standard procedure, such as microbial culture.

## Figures and Tables

**Table 1:** Characteristics of Human Subjects.

			All (n = 334)	
Age (mean ± SD), in years			35.8 (±15.2)	
Time to obtain result			20 (±) seconds	
Sex (%)				
Male			187 (55.99%)	
Female			147 (44.01%)	
			Positive	Negative
Eligible participants			139 (41.6%)	195 (58.4%)
		Lesion absent	Lesion present	X-ray not done
Chest X-ray status		186 (55.68%)	103 (30.83%)	45 (13.47%)
		Positive	Negative	Not known
HIV status		21 (6.28%)	212 (63.47%)	101 (30.23%)
		Diabetic	Not diabetic	Not known
Diabetic status		21 (6.28%)	209 (62.57%)	104 (31.13%)
		Current smoker	Past smoker	Never smoked
Smoker status		34 (10.17%)	52 (15.56%)	248 (74.25%)

**Table 2:** Confusion Matrix.

	Classification According to Breath Test			
		Positive	Negative	Total
Disease Status	Tuberculosis	TP (133)	FN (6)	139
	Healthy Control	FP (17)	TN (178)	195
Total		150	184	334

TP = true positive, TN = true negative, FP = false positive, and FN = false negative.

**Table 3:** Result of Diagnostic accuracy Tests.

Total TP = 133 FP = 17 FN = 6 TN = 178	n	Sensitivity (95% CI)	Specificity (95% CI)	PPV	NPV	ROC Area
	334	95.7% (90.8–98.4)	91.3% (86.4–94.8)	88.7%	96.7%	0.935
Age group (15–29) TP = 68 FP = 8 FN = 2 TN = 70	148	97.1% (90.1–99.7)	89.7% (80.8–95.5)	89.5%	97.2%	0.934
Age group (30–44) TP = 33 FP = 6 FN = 4 TN = 52	95	89.2% (74.6–97)	89.7% (78.8–96.1)	84.6%	92.9%	0.894
Age group (45–59) TP = 17 FP = 2 FN = 0 TN = 39	58	100% (80.5–100)	95.1% (83.5–99.4)	89.5%	100%	0.976

PPV = positive predictive value, NPV = negative predictive value, and ROC = receiver operating characteristic curve.

## Data Availability

The data presented in this study are available on request from the corresponding authors.
